# Ca^2+^ activity in the HSN egg-laying command neurons and animal age is accompanied by a delay in the defecation motor program in *Caenorhabditis elegans* (I)

**DOI:** 10.17912/micropub.biology.000093

**Published:** 2019-03-29

**Authors:** Bhavya Ravi, Kevin M. Collins

**Affiliations:** 1 Neuroscience Program, University of Miami Miller School of Medicine, Miami, FL 33136; 2 Department of Biology, University of Miami, Coral Gables, FL 33146; 3 Present address: Department of Neurology and Neuroscience, Johns Hopkins University School of Medicine, Baltimore, MD 21205

**Figure 1:**
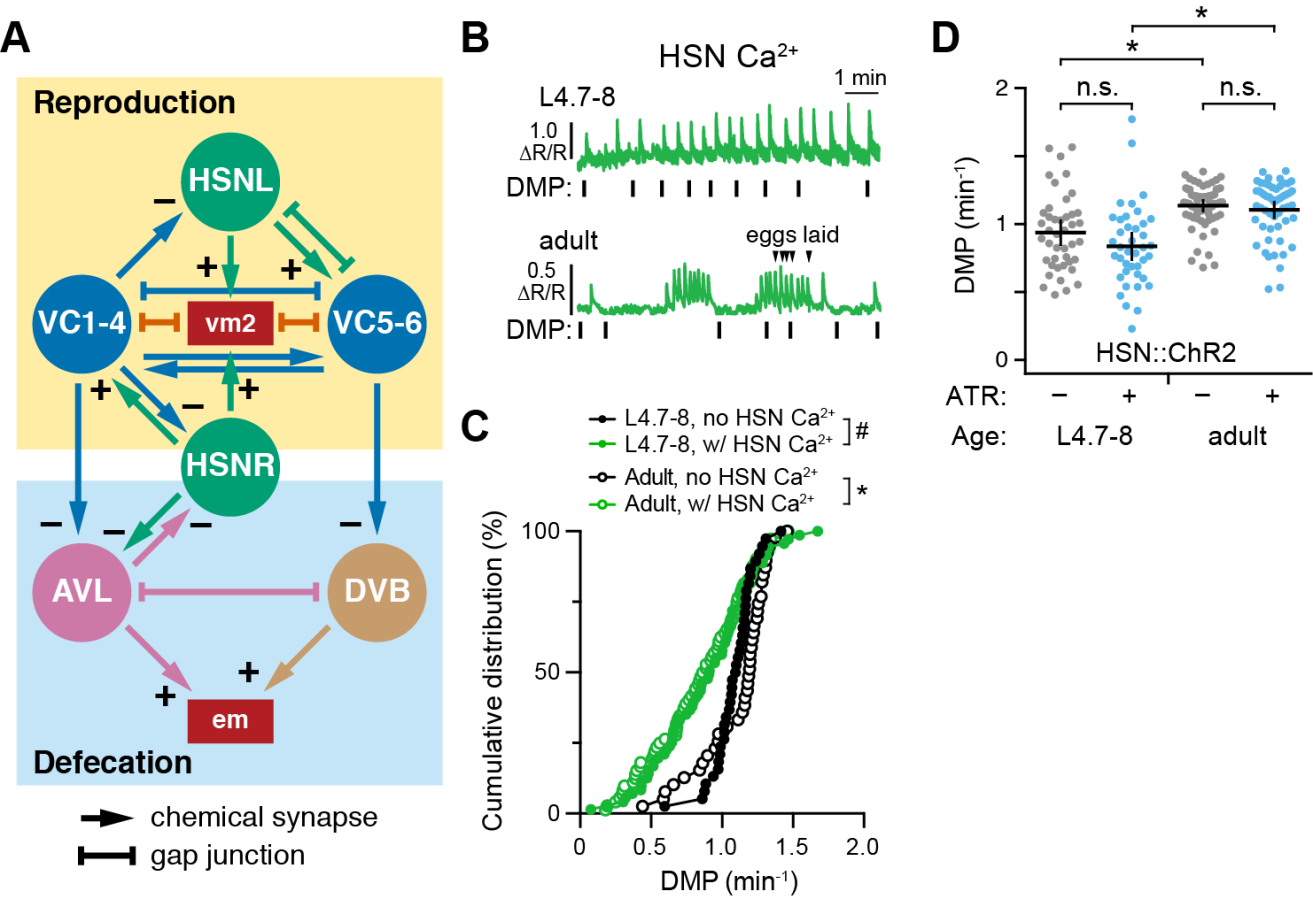
(**A**) Wiring diagrams of the reproductive circuit (top) and defecation motor circuit (bottom). HSN (green) and VC (blue) neurons synapse onto each other and the vm2 muscles for egg laying. Data from White J.G. et al. (1986) indicate HSN and VC also make and receive synapses from AVL and DVB, excitatory GABA motor neurons that regulate the contraction of the enteric muscles (em) for defecation. Arrows indicate chemical synapses, and + or – indicates a presumptive excitatory or inhibitory synapse, respectively. Bar-headed lines indicate gap junctions (e.g. electrical synapses). (**B**) Representative HSN Ca^2+^ traces at the L4.7-8 larval stage (top) and adults (bottom). Vertical lines indicate the expulsion step of the defecation motor program (DMP); arrowheads indicate adult egg-laying events. (**C**) Cumulative distribution plots showing instantaneous frequency of the DMP events (min^-1^) with no observed HSN Ca^2+^ transient (black) and those with one or more HSN Ca^2+^ transients (green) in L4.7-8 (closed circles) and adult animals (open circles). Pound indicates p=0.0058; asterisk indicates p<0.0001 (Kruskal-Wallis test with Dunn’s correction for multiple comparisons). Total DMP intervals used for analysis: L4.7-8 DMPs without an HSN Ca^2+^ transient (n=33); L4.7-8 DMPS with at least one HSN Ca^2+^ transient (n=62) from 9 animals; Adult DMPs without an HSN Ca^2+^ transient (n=39); Adult DMPs with at least one HSN Ca^2+^ transient (n=72) from 11 animals. (**D**) Scatterplots showing the consequences of HSN optogenetic activation on DMP frequency. L4.7-8 and adult animals expressing Channelrhodpsin-2 (ChR2) in HSN neurons from the *wzIs30* transgene were grown in the absence (–, grey) or presence (+, blue) of all-*trans* retinal (ATR), illuminated with continuous blue light for two minutes, and the timing of DMP events was recorded. The elapsed time between Expulsion events was used to calculate an instantaneous DMP frequency from each recorded interval (min^-1^) from X animals. Error bars show 95% confidence intervals for the mean; * indicates p<0.0001; n.s. indicates p=0.2102 (L4.7-8) or p>0.999 (Adult); one-way ANOVA with Bonferroni correction for multiple comparisons. Total DMP intervals used for analysis from 10 animals: L4.7-8, no ATR (n=43); L4.7-8, plus ATR (n=40); Adult, no ATR (n=67); Adult, plus ATR (n=55).

## Description

We have recently described an unusual rhythmic Ca^2+^ activity rhythm in the developing Hermaphrodite Specific Neurons (Ravi *et al.* 2018b). This ~50 s rhythm of HSN activity in L4.9 animals resembled the rhythm of the defecation motor program (DMP), prompting us to investigate whether there is a relationship between circuits that regulate reproduction and defecation behaviors. As shown in Figure 1A, the egg-laying HSN command neurons and VC motor neurons make and receive synapses from the excitatory GABAergic AVL and DVB motoneurons that regulate defecation (White, J.G. *et al.* 1986). Serotonin and Gα_o_ signaling, which regulate egg laying behavior, can also signal to inhibit defecation (Ségalat *et al.* 1995; Waggoner *et al.* 1998; Hardaker *et al.* 2001; Tanis *et al.* 2008; Brewer *et al.* 2019). However, the functional relationship between what are thought to be independent motor circuits has not been examined. Because evidence shows that both the egg-laying active state and the DMP are both linked to changes in forward and reverse locomotion (Hardaker *et al.* 2001; Nagy *et al.* 2015), we reasoned there may be a similar relationship between expulsive behaviors that drive either egg laying or defecation.

Using a transgene that co-expresses GCaMP5 and mCherry in the HSNs from the *nlp-3* promoter (Collins *et al.* 2016), we performed ratiometric Ca^2+^ imaging in L4.7-8 juveniles and egg-laying adults and compared the timing of HSN Ca^2+^ transients and defecation events (Ravi *et al.* 2018a). We found that defecation intervals in L4.7-8 and adult animals were significantly longer when they were accompanied by one or more HSN Ca^2+^ transients (Fig. 1B and 1C). This suggested the HSNs might signal to inhibit the defecation motor rhythm. To test this, we used a transgene that expressed Channelrhodopsin-2 in the HSNs from the *egl-6* promoter (Emtage *et al.* 2012) and tested whether acute optogenetic activation of the HSNs in L4.7-8 juveniles or adults affected the DMP rhythm. Blue light illumination of animals grown on ATR, an essential cofactor for ChR2, caused a mild reduction in DMP frequency in L4.7-8 animals, but this effect was not statistically significant (p=0.2102) and was not observed in adults (Fig. 1D). Interestingly, we observed that DMP frequency was significantly longer in 24-hour adult animals compared to L4.7-8 juveniles (Fig. 1D). Previous work has shown a significant decline in the DMP frequency in aging animals, although this is reduction was not apparently related to changes in feeding as measured by pharyngeal pumping (Croll *et al.* 1977; Bolanowski *et al.* 1981). We propose that these changes in defecation frequency may relate to onset of egg-laying behavior and/or continued growth of adult animals.

## Reagents

Strains are available from CGC: LX2004 *vsIs183* [*nlp-3p::GCaMP5::nlp-3* 3’UTR + *nlp-3p::mCherry::nlp-3* 3’UTR + *lin-15(+)*] *lite-1(ce314)* *lin-15(n765ts) X*; LX1836 *wzIs30* [*egl-6::ChR2-YFP::unc-54* 3’UTR + *lin-15(+)*] IV, *lite-1(ce314)* *lin-15(n765ts) X*; All-*trans* retinal (100 mM stock in ethanol) was from Sigma and added to pre-warmed OP50 bacterial cultures in B Broth as described (Collins *et al.* 2016; Ravi *et al.* 2018b). DMP frequency was measured based on the timing of the final expulsion step (Liu and Thomas 1994). Ratiometric Ca^2+^ imaging was performed in freely behaving animals as previously described (Collins *et al.* 2016; Ravi *et al.* 2018a; b).
